# The Importance and Place of Methemoglobin and Carboxyhemoglobin Levels in the Diagnosis and Prognosis of Pulmonary Embolism

**Published:** 2019-01

**Authors:** Fatih Uzer, Omer Ozbudak

**Affiliations:** 1 Kastamonu State Hospital, Department of Respiratory Medicine, Kastamonu, Turkey; 2 Akdeniz University Faculty of Medicine, Department of Respiratory Medicine, Antalya, Turkey

**Keywords:** Carboxyhemoglobinemia, Methemoglobinemia, Pulmonary embolism

## Abstract

**Background:**

This is a retrospective study to investigate the effects of Carboxyhemoglobin (COHb) and Methemoglobin (MetHb) levels in the diagnosis and prognosis of Pulmonary Thromboembolism (PTE).

**Materials and Methods:**

Cases that were confirmed with PTE diagnosis using CT Pulmonary Angiography (CTPA) or Ventilation/Perfusion Scintigraphy were accepted as pulmonary embolism. And patients which were excluded using the same methods were accepted as the control group. Patients with carbon monoxide poisoning, Chronic Obstructive Pulmonary Disease (COPD), sepsis, pneumonia, asthma, idiopathic pulmonary fibrosis, bronchiectasis, decompensated cardiac failure or those who used drugs that cause methemoglobinemia (sulphanomides, dapson, phenacetin, primacine, benzocaine) were not included in the study.

**Results:**

In our study, 462 patients were examined with an initial PTE diagnosis. Among these patients, 107 patients who met the inclusion criteria were included in the study. The mean age of all patients was 56.44 ±17.3 years (21–86) and the mean age of patients with PTE diagnosis was 55.3 years and the mean age of excluded patients was 59 years (p:0.27). When the blood gas parameters of both groups were compared, COHb levels in the groups with PTE diagnosis were statistically significantly higher (p=0.001), and the PO_2_ levels in the group excluded for PTE diagnosis were statistically significantly higher (p=0.028). In our study, six of our patients (8.1%) died in the early stages because of PTE.

**Conclusion:**

In our study, COHb level was found to be statistically significant in the group with PTE. However, this value was not higher than the normal COHb level in the blood. We found that MetHb and COHb levels were not statistically significant in the prognosis of PTE.

## INTRODUCTION

Pulmonary Thromboembolism (PTE) can be defined as blocking of the pulmonary artery or one of its branches with materials (thrombus, tumor, air or fat) from another part of the body (1–3). PTE is a disease that is commonly seen in the chest diseases practice and has a mortality rate of 10–15% within the first three months period after the diagnosis (4,5). There is no single laboratory marker for definite diagnosis of PTE. Therefore, studies are mostly focused on an ideal marker that provides a diagnosis. Ideal prognostic and diagnostic markers should be easily evaluated and tested. Methemoglobin (MetHb) and Carboxyhemoglobin (COHb) are values that can easily be measured in the blood gas.

Carbon monoxide (CO) can bind to the place in the hemoglobin (Hb) molecule that oxygen binds and thus remove oxygen from hemoglobin (6). Increased exhaled CO is seen in inflammatory pulmonary diseases such as bronchiectasis and upper respiratory infection (7). Studies have shown that COHb amount could be related with the severity of acute ischemic diseases (8). Increased COHb levels have been reported for critical diseases including PTE (7,9). CO is produced endogenously by the heme oxygenase enzyme group. Heme oxygenase enzyme is stimulated with cytokines and Nitric Oxide (NO). In hemoglobin catabolism 3 enzymes act such as; hemoxgenase-1, hemoxygenase-2 and hemoxygenase-3 (7,9–11). Hemoxgenase-1 (HO-1); can be induced by biochemical or biophysical stress and is the common isoform of the enzyme (12). It is densely present in vascular endothelium, smooth muscle, spleen and liver. Most known stimulators of HO-1 are; heme and heme derivatives, heat shock, heavy metals, NO, NO donors, oxygenated lipids, hyperoxygenemia, oxidative stress, cytokines, strong light, and glucose absence (7,9–13). Increase in HO-1 is a sign of oxidative stress as a general indicator (10).

In order for hemoglobin to bind reversibly with oxygen and carry oxygen to tissues, iron should be kept in ferric (Fe 2+) form. Hemoglobin in Ferric (Fe 3+) form which has oxidized iron atom and is unable to bind oxygen is called MetHb (14–17). In some medical conditions such as painful attacks that are seen in sepsis, gastrointestinal infections and sickle cell anemia, MetHb could occur due to contact with toxic agents and chemical substances. Hypoxia and oxidative stress are known to increase MetHb formation (15–19). Nitric Oxide Synthase (NOS) activation, hypoxemia and oxidative stress due to PTE could cause endogenous increase in MetHb (14–16).

Our objective was to investigate whether COHb and MetHb levels were contributed to PTE diagnosis and prognosis in patients who were examined for PTE in our center.

## MATERIALS AND METHODS

This is a retrospective study to investigate the effects of COHb and MetHb levels in the diagnosis and prognosis of pulmonary thromboembolism. Patients suspected of PTE who presented to the Chest Diseases Outpatient Clinic and Emergency Department of the School of Medicine of Akdeniz University between 01.11.2015 and 31.08.2016 were included in the study.

Patients admitted to Chest Diseases Outpatient Clinic and Emergency Department with complaints of chest pain, shortness of breath, palpitation or having PTE pre-diagnosis by clinical and laboratory signs were recorded in our hospital automation system. Afterwards diagnosis was confirmed with related diagnostic procedures or excluded. The name and protocol numbers of the patients with diagnosis code I26 (Pulmonary embolism), I26.0 (Pulmonary embolism together with acute cor pulmonale), I26.9 (pulmonary embolism, without acute cor pulmonale) from the hospital automation system were recorded. Demographic characteristics and clinical information of patients were collected twice in our hospital. We examined both forms of medical records (handwritten and electronic). In the event of unrecorded data or if any discrepancy appeared in either one of the sources, the respective case was rejected.

The patients were divided into two groups. Cases that were confirmed with PTE diagnosis using CT Pulmonary Angiography (CTPA) or Ventilation/Perfusion Scintigraphy were accepted as pulmonary embolism. And the patients which were excluded using the same methods were accepted as the control group. CTPA and V/P scintigraphy reports were given by expert radiologists.

Patients with CO poisoning, Chronic Obstructive Pulmonary Disease (COPD), sepsis, pneumonia, asthma, idiopathic pulmonary fibrosis, bronchiectasis, decompensated cardiac failure which could cause carboxyhemoglobinemia or those who used drugs that caused methemoglobinemia (sulfonamides, dapsone, phenacetin, primaquine, benzocaine), positive smoking status at the time of admission and absence of blood gas analysis on arrival were not included in the study. The diagnosis of COPD was ruled out by pulmonary function test.

Well’s score and Pulmonary Embolism Severity Index (PESI) at the time of arrival was calculated based on the appropriate baseline clinical, demographic, and serologic characteristics of each patient using the values retrieved from the computerized medical records system.

Demographic and clinical characteristics including age, heart rate, respiratory rate, systolic blood pressure, and diastolic blood pressure of all patients were recorded. D-dimer, troponin, Brain Natriuretic Peptide (BNP), creatinine levels, and full blood counts were entered in data form. D-dimer level > 0.55 mg/dl, BNP level 100 mg/dl and troponin level >0.01 mg/dl was accepted as significant. Arterial blood gas measurements taken at ambient temperature at the early stage following the complaints of patients monitored in the Emergency Department (before oxygen therapy), outpatient clinic and/or Chest diseases Department were entered in the data form. pH, PCO_2_, PO_2_, MetHb and COHb values in blood gas were recorded.

Patients diagnosed as having acute PTE were classified, based on early mortality risk, in groups of high (massive PTE), intermediate (sub-massive PTE), and low risk (non-massive). Patients with hemodynamic instability [sustained hypotension, cardiac arrest (pulselessness), and persistent symptomatic bradycardia] were considered affected by high-risk PE and therefore as candidates for thrombolytic therapy. A PESI score greater that 85 points was considered as indicative of intermediate risk in hemodynamically stable patients. Patients characterized by intermediate-risk status and at the same time imaging and biochemical findings compatible with Right Ventricular (RV) dysfunction and/or myocardial necrosis were classified as intermediate risk patients. Finally, hemodynamically stable patients with acute PTE and a PESI score less than 85, with no RV dysfunction or myocardial necrosis were considered as low risk.

### Data evaluation

Results were reported as the mean ± SD and categorical variables were given as percentages. The normality of distribution was confirmed by the Shapiro-Wilk’s **W**-test. Since Shapiro–Wilk’s test results showed the fitting of the observed data against a normal distribution, and statistical analysis of clinical data between two groups consisted of unpaired t-tests, whereas the Chi-square/Fisher’s exact tests were used for categorical variables. Analyses were performed with IBM SPSS Statistics for Windows, Version 20.0 (IBM Corp. Released 2013. Armonk, NY: IBM Corp.) software and two-tailed P value less than 0.05 was considered statistically significant.

### Ethics of the Study

The study was approved by the Ethics Board of School of Medicine of Akdeniz University.

## RESULTS

**Figure 1.**
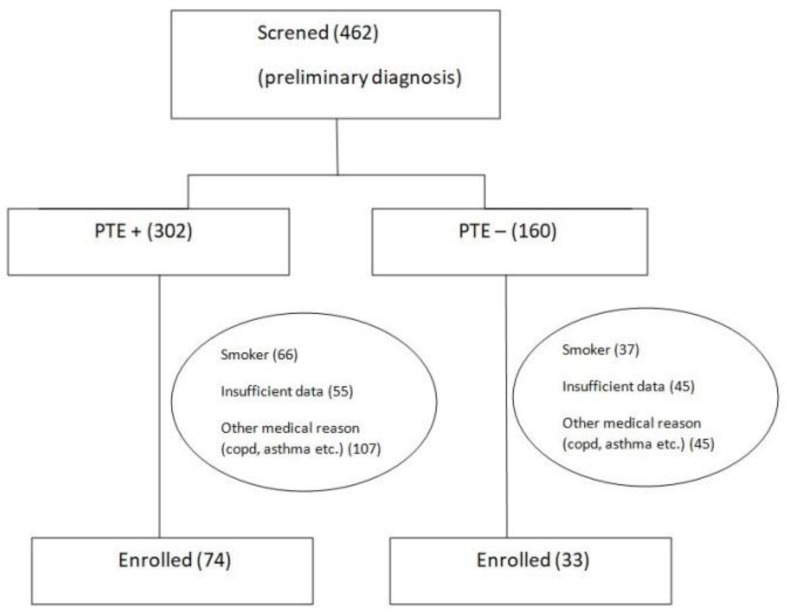
Distribution of patients according to diagnostic features.

In our study, totally 462 patients were evaluated for PTE pre-diagnosis. In 302 patients PTE diagnosis was verified, whereas in 160 patients PTE was excluded. In PTE group 228 patients and in other group 127 patients were excluded from the study because of various causes (being smoker, lack of data, comorbidities such as COPD). Finally total 107 patients having inclusion criteria were included in the study (Figure 1).

Basic clinical and laboratory characteristics of the patients enrolled in the study are shown in table 1. Seventy-four (69.2%) patients were diagnosed with PTE and 33 (30.8%) were included in the exclusion group. 56.1% of the patients were female and 47(43.9%) were male. While 32(29.90%) of the patients had a smoking history, 75 (70.10%) of the patients did not. In this study, 73 patients had at least one comorbidity and the most frequent comorbidities were hypertension (24%) and diabetes mellitus (20.6%). Fifty-nine (79.7%) of the patients diagnosed with PTE had non-massive PTE, 10 (13.51%) had sub-massive PTE and 5 (6.7%) had massive PTE. The group of patients diagnosed with PTE consisted of significantly higher number of male patients (P=0.006). The male patients diagnosed with PTE had a significantly older mean age than the female patients (P=0.014). Sociodemographics of the patients who were diagnosed with PTE, but excluded are shown in table 2.

**Table 1.** Basic clinical and laboratory characteristics of all cases

	Mean+SD	Distribution range
D-dimer (mg/dL)	2.16 ± 3.76	0.17–2.86
Troponin (ng/mL)	0.05 ± 0.18	0.00–1.74
BNP (pg/ml)	183.61 ± 571.04	0.00–500.00
Fibrinogen (mg/dL)	388.55 ± 124.00	185.00–811.00
Neutrophile (thousand/mm^3^)	6.03 ± 3.53	0.43–20.650
Lymphocyte (thousand /mm^3^)	1.95 ± 0.88	0.26–4.89
Creatinine (mg/dL)	0.94 ± 0.57	0.31–4.89
Methemoglobin	0.25%±0.16	0.00–1.00
Carboxyhemoglobin	0.79%±0.64	0.00–3.50
pH	7.43 ± 0.04	7.28–7.57
Wells	4.58 ± 1.18	3.00–8.00
PESI	74.93 ± 32.40	21.00–255.00
PO_2_ (mmHg)	79.42 ± 26.54	20–145.00
PCO_2_ (mmHg)	33.93 ± 7.32	19–64.00
EF	60.84 ± 6.52	30–70
mPAP (mmHg)	42.64 ± 15.69	25–100

SD: Standard deviation; PESI: Pulmonary embolism severity index; EF: Ejection Fraction; mPAP: Pulmonary artery pressure; BNP: Brain natriuretic peptide

**Table 2.** Sociodemographic characteristics of PTE diagnosis

		PTE Diagnosis (n, %)	No PTE Diagnosis (n, %)	P
> 60 years	Male	21 (70)	6 (30)	0.014
	Female	9 (30)	14 (70)	
Smoking history	Yes	24 (32.4)	8 (24.2)	0.393
	No	50 (67.6)	25 (75.8)	
Comorbidity	Yes	49 (66.2)	24 (72.7)	0.504
	No	25 (33.8)	9 (27.3)	

When biochemistry and hemogram results of the group diagnosed with PTE and group excluded for PTE diagnosis were compared, while fibrinogen levels (P=0.029) were significantly higher in the group diagnosed with PTE, no significant difference in other parameters was found between these two groups. When the blood gas parameters of both groups were compared, COHb levels in the groups with PTE diagnosis were statistically significantly higher (P=0.001), and the PO_2_ levels in the group excluded for PTE diagnosis were statistically significantly higher (P=0.028) (Table 3).

**Table 3.** The statistics for hemogram and biochemistry results for both groups

	PTE Diagnosis (Mean±SD)	No PTE Diagnosis (Mean±SD)	p
Fibrinogen (mg/dL)	406.05±131.62	351.23±97.76	0.029
Troponin (ng/mL)	0.05±0.09	0.07±0.30	0.192
BNP (pg/mL)	211.35±683.15	128.14±214.69	0.762
D-dimer (mg/dL)	2.48±4.33	1.45±1.86	0.211
Creatinine (mg/dL)	0.94±0.46	0.95±0.76	0.526
Neutrophile (thousand/mm^3^)	6.16±3.39	5.74±3.87	0.213
Lymphocyte (thousand/mm^3^)	2.02±0.98	1.78±0.59	0.198
PH	7.43±0.04	7.44±0.05	0.500
Methemoglobin (%)	0.25±0.17	0.26±0.16	0.747
Carboxyhemoglobin (%)	0.90±0.57	0.54±0.46	0.001
PO_2_ (mmHg)	76.07±25.54	86.94±27.60	0.028
PCO_2_ (mmHg)	33.64±6.38	34.58±9.15	0.549
EF	60.92±7.11	60.65±4.99	0.352
mPAP (mmHg)	41.13±15.34	46.42±16,25	0.089
Wells	4.84±1.15	4.00±1.06	0.001

Ef: Ejection Fraction; mPAP: Pulmonary artery pressure; BNP: Brain natriuretic peptide; PESI: Pulmonary Embolism Severity Index; SD:Standard Deviation

When sub-group analysis of patients diagnosed with PTE was made, troponin (P=0.0001), BNP (P=0.002), D-dimer (P=0.001) levels, Pulmonary Artery Pressure (mPAP) (P=0.023) and Wells (P=0.016) and PESI (P=0.001) scores in patients diagnosed with massive and sub-massive PTE were significantly higher compared to patients diagnosed with non-massive PTE. There was no significant difference between the two groups for all other parameters (Table 4).

**Table 4.** Sub-group analysis of patients diagnosed with PTE

	Non-massive (Mean±SD)	Massive+ sub-massive (Mean±SD)	p
Methemoglobin (%)	0.23±0.13	0.32±0.26	0.283
Carboxyhemoglobin (%)	0.85±0.65	1.14±0.76	0.112
Troponin (ng/mL)	0.02±0.05	0.15±0.15	0.0001
BNP (pg/mL)	205.82±769.74	229.38±261.16	0.002
Fibrinogen (mg/dL)	415.70±132.29	371.59±127.86	0.330
D-dimer (mg/dL)	1.42±1.58	6.63±8.01	0.001
PH	7.44±0.04	7.43±0.03	0.627
Wells	4.67±1.09	5.50±1.18	0.016
PESI	69.76±26.73	106.73±43.59	0.001
EF	61.14±6.80	60±8.52	0.569
mPAP (mmHg)	37.51±8.22	57.27±26.69	0.023

EF: Ejection Fraction; mPAP: Pulmonary artery pressure; BNP: Brain natriuretic peptide; PESI: Pulmonary Embolism Severity Index; SD:Standard Deviation

No statistically significant correlation was found in regard to PESI, Wells score, troponin and D-dimer levels of both COHb and MetHb in the Spearman correlation test.

In our study, six of our patients (8.1%) died in the early stages because of PTE. Four (66.6%) of the patients were male and 2 (33.3%) were female. When deceased and survived patients were compared, mean Wells and PES score, D-dimer and troponin levels were significantly higher in deceased patients. No significant difference was found in any of the investigated parameters (Table 5).

**Table 5.** Comparison of deceased and survived patients

	Survived (Mean±SD) (n:68)	Deceased (Mean±SD) (n:6)	P
Methemoglobin (%)	0.24±0.15	0.40±0.30	0.150
Carboxyhemoglobin (%)	0.87±0.65	1.2±0.95	0.200
Troponin (ng/mL)	0.04±0.85	0.12±0.16	0.028
BNP (pg/mL)	223.11±716.47	97.653±106.10	0.604
Fibrinogen (mg/dL)	410.14±135.14	366.50±89.47	0.653
D-dimer (mg/dL)	1.63±1.87	12.06 ±10.34	0.008
Wells	4.49±1.00	5.92±1.28	0.008
PESI	72.47±27.11	131.5±56.50	0.006
Age (yrs)	54±16.7	62±14.8	0.332

BNP: Brain natriuretic peptide; PESI: Pulmonary Embolism Severity Index; SD:Standard Deviation

## DISCUSSION

Pulmonary embolism is a disease, which is commonly seen in the chest diseases practice and has a mortality rate of 10–15% within the first three months period after the diagnosis (4,20). It is one of the most common causes of hospital deaths. While a significant decrease in mortality associated with clinical conditions such as stroke and acute coronary syndrome has been observed in recent years, no apparent decrease in mortality caused by PTE has been observed (21). With the increase in use of diagnostic methods, most notably CT, the diagnostic and therapeutic process of pulmonary embolism, which is frequently seen in Emergency Department changes depending on the clinical characteristics of the patient. Together with decreased mortality and morbidity and less Emergency Department visits and shorter hospital stays in Chest diseases Departments and calculation of associated costs, early discharge and outpatient treatment of patients with lower risk should be planned. It is important to have an early diagnosis and provide correct treatment for such a common and fatal disease.

There is no single laboratory marker for definite diagnosis of PTE. Therefore, studies are mostly focused on an ideal marker that provides a diagnosis. Ideal prognostic and diagnostic markers should be easily evaluated and tested. MetHb and COHb are values that can easily be measured in the blood gas.

Various biochemical markers are used for PTE diagnosis. The most known and widely used of these is D-dimer test. The level of D-dimer, which is a specific fibrin split product, can increase in Deep Vein Thrombosis (DVT), PTE patients, atherosclerotic cardiac diseases, renal diseases, mesenteric diseases, and cerebrovascular stroke (1,2,22–24). It is recommended to evaluate clinical pretest probability score and D-dimer test results together (22,23). In our study, D-dimer level in 78.89% (78) of the patients who received the test was high. Contrary to the literature, no significant difference in D-dimer levels was found between two groups. However, this was significantly high in mortality patients.

Blood gas changes such as hypoxemia, hypocapnia and respiratory alkalosis are most commonly seen in PTE patients. Additionally, normal blood gas values of 10–25% can be found (1,2,22,25,26). PO_2_ levels were significantly lower in the group with PTE diagnosis, however, no statistically significant difference was found for pH and PCO_2_ levels. In the study conducted by Masotti et al. (27), mean PO_2_ was 54 mmHg and mean PCO_2_ was 41 mmHg in patients with PTE diagnosis. These values were 76.07 mmHg and 33.64 mmHg, respectively in our study.

There are several studies that demonstrate the relationship between MetHb and COHb levels and pulmonary embolism in the literature. There was one study that we could access which was conducted retrospectively by Kakavas et al. (9) in Greece in 2015. In this study, 156 patients were diagnosed with PTE in the Emergency Department with a median age of 76 years and similar gender distribution. This is the only study in this area and the inclusion criteria were very expansive and exclusion criteria were very few. All patients that had PTE diagnosis confirmed using CTPA were included in the study; only active smokers were excluded. Therefore, pneumonia, sepsis, and acute ischemic events that could raise COHb level and conditions that could raise MetHb levels were not excluded.

In our study there were more female patients (56.1%) and the mean age was 55.3 years in patients diagnosed with PTE. In the study of Kakavas et al. when compared with parameters that show the severity and risk of the disease such as troponin, BNP and Apache II, MetHb and COHb values were found to be significant (9). However, our study found that MetHb and COHb levels did not contribute to the diagnosis and prognosis of PTE.

In our study, COHb values were significantly higher in patients diagnosed with pulmonary embolism versus patients who were excluded for pulmonary embolism. The mean COHb value in the normal healthy population is between 0.3–2.6%. Although this value was in the normal range, it was significantly higher when compared with the control group. In the analysis of pulmonary embolism subgroup, when patients with non-massive PTE diagnosis were compared with patients with massive and sub-massive PTE diagnosis, no significant difference was found between COHb levels. In the study by Kakavas et al., COHb values in patients diagnosed with high risk (massive PTE + sub-massive PTE) PTE were low, however, our study does not support this. COHb levels were low in mortality patients in Kakavas et al’s study (9). Therefore, they reported that COHb levels could be used as a marker in the prognosis of acute PTE. However, our study did not find any significant difference in COHb levels of our mortality and survived patients.

No significant difference in MetHb values was found between the group with PTE diagnosis and the group excluded for PTE diagnosis. When we look at the subgroup analysis of the group diagnosed with PTE, the low risk group and medium and high risk groups were compared and no significant difference was found for MetHb levels. Kakavas et al. reported that MetHb levels could rise in the high risk group with PTE diagnosis (9). However, our study did not find any correlation between MetHb and PTE. We believe that the reason for the positive correlation between MetHb and PESI in the study of Kakavas et al. was the extensive inclusion criteria.

One of the three clinical conditions; massive, sub-massive and non-massive is found in PTE. Massive PE is seen in 4.2% of all PE cases and has a mortality rate of 58% (4). In our study 5 (6.75%) of the patients had massive, 10 (13.51%) had sub-massive, and 59 (79.72%) had non-massive PTE. When we look at its effect on mortality of the patients, 3 (60%) had massive PTE diagnosis, and the other 3 (22.20%) had sub-massive PTE diagnosis. As expected mortality rates were higher in massive and sub-massive patients.

High BNP and troponin values which are markers of RV dysfunction are associated with early mortality and complicated clinical course (1,22, 23). In our study, no statistically significant difference was found in BNP and troponin levels between the patients who were diagnosed with PTE and who were excluded for PTE diagnosis, however, when massive and sub-massive PTE and non-massive PTE was compared, troponin levels were significantly higher in the massive and sub-massive group. In their study, Kakavas et al. found a negative correlation between COHb and NT-proBNP and troponin and a positive correlation between MetHb and NT-proBNP and troponin (9). Since a correlation was found between COHb and MetHb levels and RV dysfunction markers, COHb and MetHb levels can be used as a marker for acute PTE prognosis. However, in our study no correlation was found between MetHb and COHb levels and RV dysfunction markers. We believe that this difference was due to the fact that conditions such as congestive heart failure, pneumonia, interstitial pulmonary diseases and acute coronary syndrome were not excluded from Kakavas et al. study.

Categorization of patients with PTE diagnosis as high risk (massive), medium risk (sub-massive) or low risk (non-massive) patients for early mortality will determine treatment options (anticoagulants/thrombolytic) and prognosis. Several clinical scoring methods are used for prognostic evaluation. The most commonly used is PESI (22). Using 11 clinical parameters that are routinely obtained in this scoring, clinicians could determine bedside PTE risks without the need for any imaging or laboratory tests. Six (8.10%) patients died because of PTE in our study. The mean PESI score of the patients was 122.5. In Kakavas et al. study a negative correlation was found between COHb and PESI and a positive correlation was found between MetHb and PESI (9). In our study, no positive or negative correlation was found between PESI score and MetHb and COHb levels.

RV dysfunction is an important prognostic indicator of PTE. Although echocardiogram (ECHO) is not routinely used in PTE diagnosis, it can be used for unstable patients who present with hypotensive shock, since it can show thrombus in right cardiac cavity and right cardiac failure (1,22, 23). There was no difference in EF level of the two groups in our study due to the study design; however, all patients that died had RV dysfunction. We believe that the reason for the lack of difference in the Pulmonary Artery Pressure (PAB) values of patients as measured with ECHO in our study is the relatively high number of patients monitored in the outpatient clinic and that ECHO tests which were done mostly in the stable stage rather than the acute stage of the disease.

In literature Schuerholz et al. (18) found that MetHb levels were associated with severity of sepsis. Yasuda et al. (7,28) reported that COHb levels were higher in asthma, COPD and idiopathic pulmonary fibrosis than control group. Mall et al. (8) found that COHb was higher in ischemic heart diseases. As a result of these studies COHb and MetHb levels were described as being associated with severity of disease. In our study in PTE (in contrast to other lung diseases) COHb and MetHb levels were not correlated with disease severity.

Our study had its own limitations. The main limitation of this study is its retrospective observational design. Consequently, the data of a few patients could not be retrieved. However, it was possible to verify the data included in our study from different sources (medical records hand written and electronic), and therefore, we consider them to be adequately precise. An additional limitation was the small number of patients in each subgroup that made statistical analysis difficult. Thus, our results should be interpreted with caution.

## CONCLUSION

Consequently PTE is a frequent disease that could result in mortality. In our study, COHb level was found to be statistically significant in the group with PTE. However, this value was not higher than the normal COHb level in the blood. Different from the only study in the literature, we found that MetHb and COHb levels were not statistically significant in the prognosis of PTE. We think that this difference is caused by the characteristics of the patient population. We believe that, this should be supported with prospective studies.
